# Nonlinear dose–response relationship between serum ferritin and anemia in Chinese children: a matched case-control study

**DOI:** 10.3389/fnut.2026.1838727

**Published:** 2026-06-12

**Authors:** Yan Sun, Yi-ya Liu, Hong-bo Li, Lin-juan He, Sheng-nan Wu, Yi-yanwen Huang, Shu Zhu, Hua Guo

**Affiliations:** Institute of Public Health Surveillance and Evaluation, Guizhou Center for Disease Control and Prevention, Guiyang, China

**Keywords:** anemia, children, matched design, serum ferritin, sex difference

## Abstract

**Objectives:**

This study aimed to investigate the dose–response relationship between serum ferritin (SF) and anemia risk in Chinese children and adolescents, with a specific focus on potential non-linearity and effect modification by sex.

**Methods:**

A 1:3 age- and sex-matched case–control study was conducted, comprising 175 anemia cases and 525 controls selected from a national school-based surveillance system. Anemia was diagnosed using WHO altitude-adjusted hemoglobin cutoffs. SF was measured by electrochemiluminescence immunoassay. The association was analyzed using unconditional logistic regression, with adjustment for confounders (age, sex, BMI, C-reactive protein) guided by a directed acyclic graph. The dose–response shape was modeled with restricted cubic splines (RCS), and a sex-SF interaction term was tested. Model performance was evaluated by the area under the ROC curve (AUC), calibration plot, and Brier score.

**Results:**

In the fully adjusted model, compared to the lowest quartile (Q1), the second (Q2: OR = 0.39, 95% CI: 0.23–0.66) and fourth (Q4: OR = 0.51, 95% CI: 0.30–0.85) quartiles of SF were associated with a significantly lower anemia risk, with a significant inverse linear trend (*P*-trend = 0.011). RCS analysis confirmed a significant non-linear relationship (*P*-nonlinearity <0.001). A significant sex interaction was observed (*P*-interaction = 0.006). Stratified analysis revealed a strong inverse association in females (e.g., Q4 vs. Q1: OR = 0.36, 95% CI: 0.18–0.73) but no significant association in males. The prediction model incorporating SF, age, sex, BMI, and CRP had an AUC of 0.61 and demonstrated good calibration (Brier score = 0.182; Hosmer-Lemeshow *p* = 0.358).

**Conclusion:**

SF exhibits a non-linear, inverse association with anemia risk in Chinese children and adolescents, which is significantly modified by sex, being pronounced only in females. These findings highlight the importance of considering both non-linear dynamics and sex-specific factors in the assessment and management of pediatric anemia.

## 1 Introduction

Anemia remains a major global public health concern, affecting approximately one-third of the world’s population, with a disproportionately high burden among children and adolescents in resource-limited settings (1). Anemia is primarily caused by a reduction in the body’s red cell volume (RCV). When red cell volume falls below the lower limit of the normal range, red blood cells cannot deliver sufficient oxygen to body tissues, thereby triggering a series of symptoms. During critical developmental periods, anemia can impair cognitive function, physical growth, and immune competence, with potential long-term consequences for health and productivity (2, 3). In China, despite significant progress, anemia persists as a notable nutritional issue within the pediatric population (4). Iron metabolism is central to erythropoiesis, and its deficiency is a leading cause of anemia worldwide (5). Serum ferritin (SF) is a key biomarker reflecting body iron stores and is routinely used to assess iron status (6). However, the biological role of SF is dual in nature: while low levels unequivocally indicate depleted iron stores, elevated SF can signal either iron overload or, more commonly in populations, the presence of subclinical inflammation, as it is a positive acute-phase reactan (7). This complexity suggests that the relationship between SF and anemia risk may not be linear or monotonic across its entire physiological and pathological range. Traditionally, epidemiological and clinical studies have focused on the risks associated with iron deficiency (low SF) (8). Emerging evidence, however, hints at a more nuanced, non-linear association. For instance, both low and high levels of iron-related biomarkers have been linked to adverse health outcomes in other contexts (9, 10). Furthermore, sex differences in iron metabolism—driven by menarche, hormonal variations, and differing dietary patterns—are well-documented (11, 12). Yet, whether sex modifies the association between SF and anemia risk in children and adolescents is poorly characterized, particularly in East Asian populations.

Therefore, significant knowledge gaps exist regarding the dose–response relationship across the full spectrum of SF levels and the potential effect modification by sex in the pediatric population. Clarifying this relationship is crucial for refining clinical interpretation of SF values and informing targeted public health strategies. To address these gaps, we conducted a 1:3 age- and sex-matched case–control study among Chinese children and adolescents. This study aimed to investigate the shape of the dose–response relationship between SF and anemia risk using non-linear modeling techniques, to quantify the association across SF quartiles, and to formally test whether this association is modified by sex.

## 2 Materials and methods

### 2.1 Study population

Based on the National Nutrition and Health Surveillance System for Students (NNHSS), this cross-sectional study investigated the association between SF and anemia among children and adolescents. The NNHSS is a national monitoring program designed to assess the implementation of the Nutrition Improvement Plan for Rural Compulsory Education Students in China. Participants were students aged 6–18 years recruited from rural primary and junior high schools in eight key surveillance counties during 2021. A multistage stratified cluster random sampling method was used. First, counties were stratified into three socioeconomic levels based on regional development indices. Within each stratum, four primary schools were selected using probability proportional to size (PPS) sampling, where the selection probability for each school was determined by its student enrollment. Subsequently, 40–60 students in grades 3–9 were randomly selected from each sampled school. Throughout this process, students with congenital diseases, severe acute infections, or incomplete key biomarker data (including hemoglobin or SF) were excluded. From this sampling frame, a 1:3 age- and sex-matched case–control analysis was conducted, resulting in a final analytic sample of 700 participants (175 anemia cases and 525 matched controls). All participants completed standardized questionnaires, physical examinations, and venous blood collection. The study protocol was approved by the Institutional Review Board of the Institute of Nutritional Health, Chinese Center for Disease Control and Prevention (No. 2021–018). Written informed consent was obtained from each participant’s guardian and from students themselves when appropriate.

### 2.2 Case-control matching strategy

**Figure 1:**
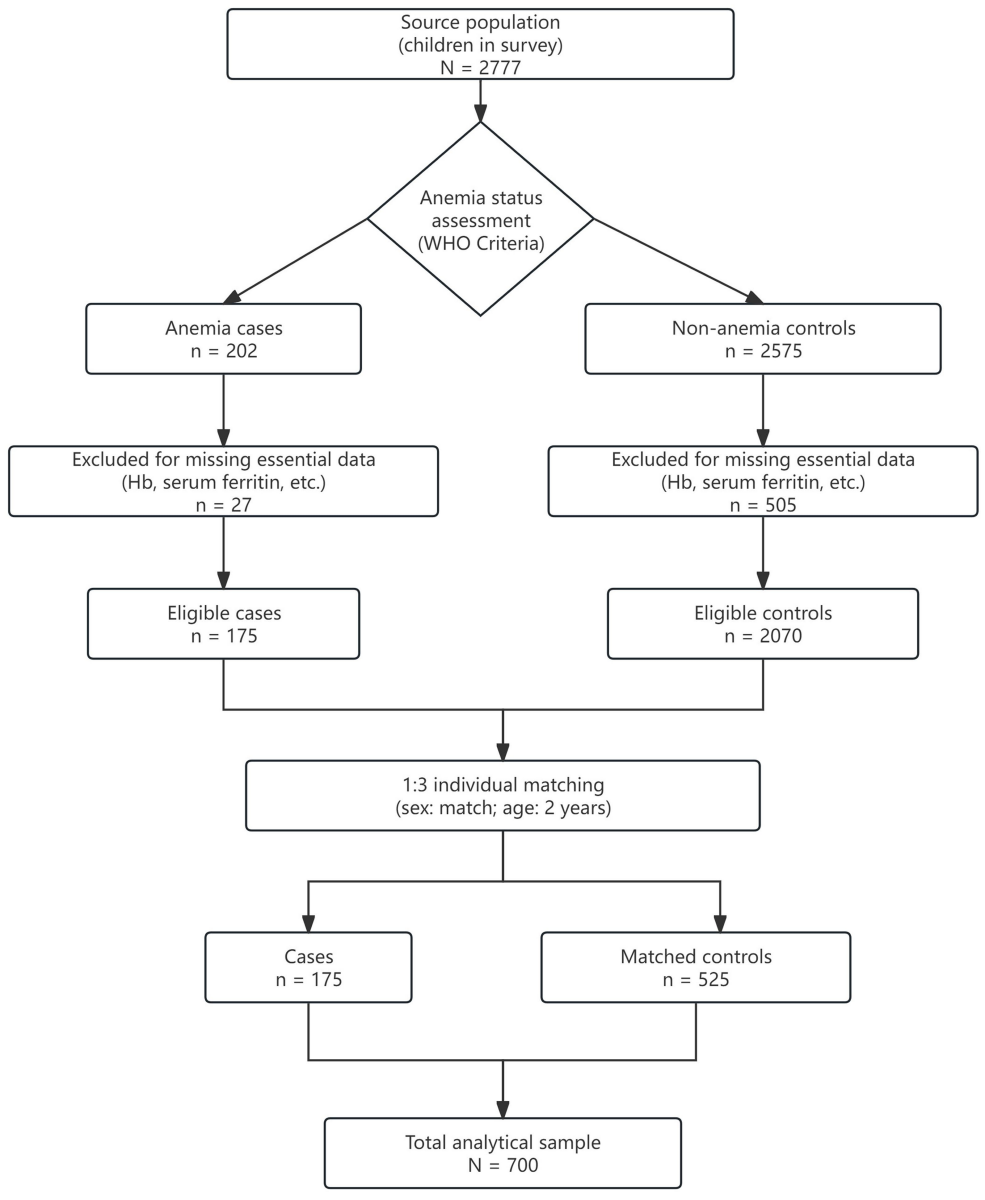
Participant selection flowchart.

A case–control analysis with 1:3 individual matching was performed. From the source population of 2,777 children and adolescents aged 6–18 years in the surveillance system, 202 children were initially identified as anemia based on the WHO hemoglobin criteria. After excluding 27 individuals with missing essential data (hemoglobin, serum ferritin, etc.), 175 eligible cases remained. From the remaining non-anemia participants (2,575 individuals), 505 were excluded due to missing essential data, leaving 2,070 eligible controls. Matching was based on sex and age within a ± 2-year range. This matching procedure yielded a final analytical sample of 700 participants, comprising 175 cases and 525 matched controls (Figure 1).

### 2.3 Clinical examination

Physical examinations were performed in the early morning under fasting conditions. Body weight was measured to the nearest 0.1 kg using a calibrated electronic or lever scale. Height was measured to the nearest 0.1 cm with a portable stadiometer. Body mass index (BMI) was subsequently calculated as weight in kilograms divided by height in meters squared (kg/m^2^) (13).

### 2.4 Blood sample determination

Fasting venous blood (5 mL) was collected from each participant. The samples were centrifuged at room temperature (3,000 rpm for 10 min, radius: 9 cm) to separate serum, which was then protected from light and transported under cold-chain conditions to designated central laboratories at the county level. SF was measured by electrochemiluminescence immunoassay. C-reactive protein (CRP) levels were determined using immunoturbidimetry. Hemoglobin concentration was assessed using a HemoCue^®^ Hb 201 + analyzer (HemoCue AB, Sweden).

### 2.5 Diagnosis of anemia

Hemoglobin (Hb) concentration was determined in venous blood samples. Anemia was defined based on age- and sex-specific cutoff values recommended by the World Health Organization (WHO) for venous blood (14). In accordance with WHO guidelines, all Hb values were corrected for altitude due to the study region’s average elevation exceeding 1,000 meters. The diagnostic thresholds applied were as follows: Hb < 115 g/L for children aged 5–11 years, Hb < 120 g/L for those aged 12–14 years, Hb < 130 g/L for males aged ≥15 years, and Hb < 120 g/L for females (non-pregnant) aged ≥15 years.

### 2.6 Statistical analysis

**Figure 2:**
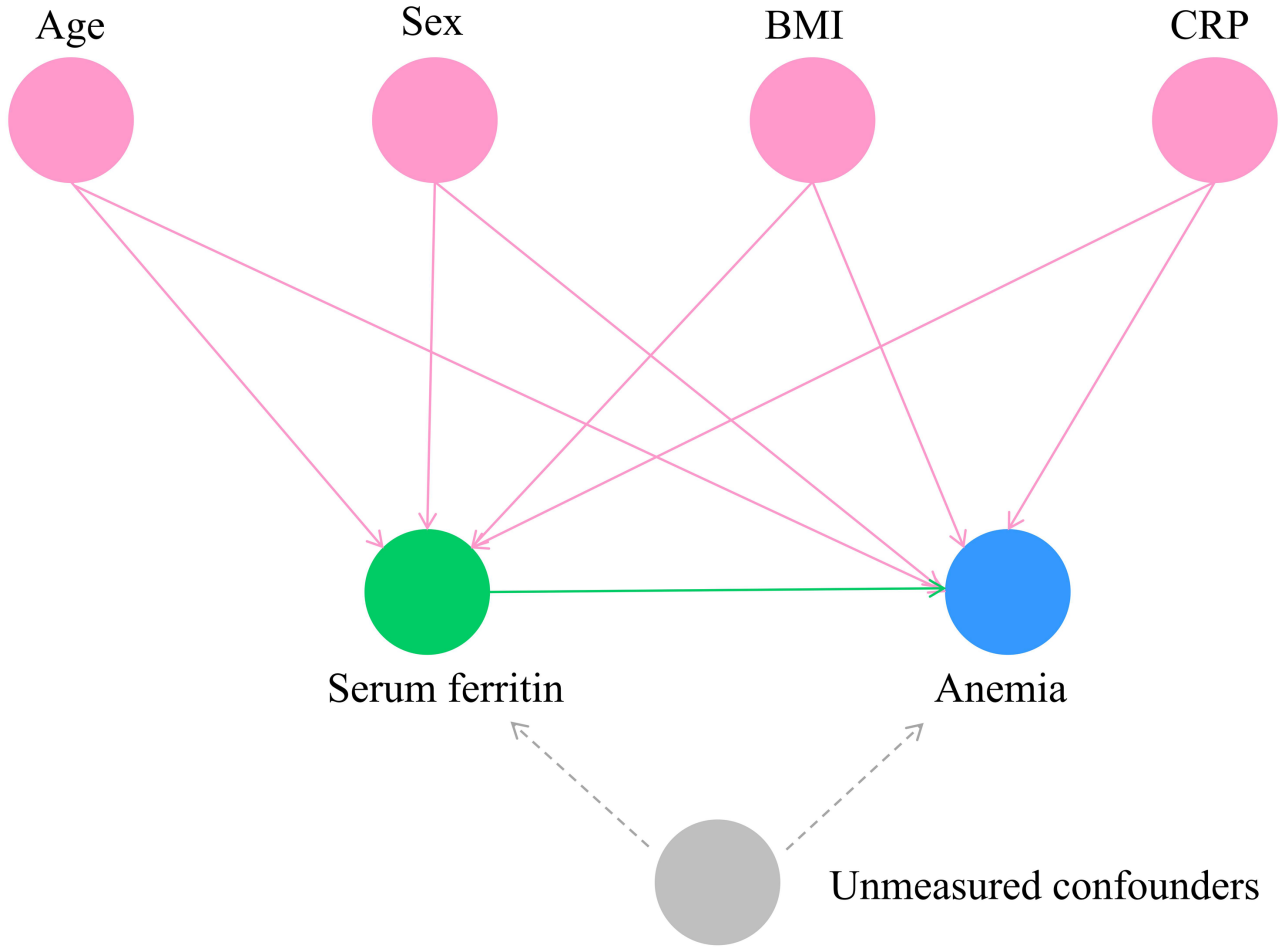
Directed acyclic graph (DAG) of the hypothesized causal relationships between SF and anemia. Green circles represent the exposure (SF), blue circles represent the outcome (anemia), and pink circles represent confounders (age, sex, BMI, CRP). Grey circles denote unmeasured confounders (e.g., dietary iron intake, menstrual status, pubertal stage). Green arrows denote the causal path (SF → anemia), pink arrows denote biasing paths (confounder–exposure and confounder–outcome), and grey dashed arrows denote potential biasing paths from unmeasured confounders to both exposure and outcome. The minimally sufficient adjustment set, based on the DAG, consists of age, sex, BMI, and CRP.

The normality of continuous variables was assessed using the Shapiro–Wilk test. As the data violated the normality assumption, they are presented as median (interquartile range). Intergroup comparisons were performed using the Mann–Whitney U test. Categorical variables are expressed as frequencies or percentages. The chi-square test was used to compare differences in the distribution of categorical variables between groups. The success of the 1:3 matched design (on sex and age ±2 years) was evaluated by calculating standardized mean differences (SMD) for all baseline variables. An absolute SMD < 0.1 was considered indicative of good balance, which was achieved for the matching factors (sex: SMD = 0.000; age: SMD = 0.005). Unconditional Logistic regression models were used to assess the association between SF and anemia. To control for potential confounding factors, matching variables (sex and age) were included as covariates in the regression model. To establish a stringent and minimally sufficient set of adjustment variables while avoiding over-adjustment bias, variable selection was guided by a directed acyclic graph (DAG) constructed based on prior literature and biological plausibility (Figure 2). This DAG model identified age, sex, BMI, and C-reactive protein (CRP) concentration as the minimal sufficient set of adjustment variables for assessing the overall effect of SF on anemia. Odds ratios (ORs) and 95% confidence intervals (CIs) were reported for each quartile increase in SF. The dose–response relationship between SF and anemia risk was modeled using restricted cubic splines (RCS). Following standard epidemiological practice, we placed five internal knots at the 12.5th, 20th, 40th, 60th, and 80th percentiles of the SF distribution. This approach, based on the widely established convention of positioning knots at regularly spaced quantiles of the exposure variable, ensures adequate flexibility for modeling non-linear patterns across the entire physiological spectrum while maintaining stability at the tails. The model used the 12.5th percentile (28.95 μg/L) as the reference point, a level near the lower end of the SF distribution that corresponds to a low iron storage state where anemia risk is anticipated to be highest. This choice provides a clinically meaningful baseline for interpreting ORs at higher SF levels. The variance inflation factor (VIF) for each covariate was below 2, indicating no substantial multicollinearity. A formal comparison between the RCS model and the linear model was conducted using the likelihood ratio test to examine the nonlinear relationship. The results revealed a statistically significant difference between the two models (*p* < 0.05), supporting the adoption of the nonlinear model. A sex-by-SF interaction was tested by including a multiplicative interaction term in the logistic regression model and evaluating its significance using the Wald test. Stratified analyses were subsequently performed. Model performance was assessed by area under the ROC curve (AUC), calibration plots, Hosmer-Lemeshow test, and Brier score. Analyses used SAS9.4 and R4.5.2 with two-sided *α* = 0.05.

## 3 Results

### 3.1 Characteristics of the study population

A total of 700 Chinese children and adolescents were included in this 1:3 matched analysis, comprising 175 anemia cases and 525 age- and sex-matched controls. The baseline characteristics of the study population are presented in Table 1. As expected due to the matching design, the distributions of age and sex were well-balanced between cases and controls (both *p* > 0.05). Compared with the control group, children with anemia had significantly lower median levels of SF and BMI (all *p* < 0.05). In contrast, the anemia group exhibited higher CRP levels, though the difference was not statistically significant (*p* > 0.05).

**Table 1:** Characteristics of anemia cases and matched controls.

Variable	Cases (*n* = 175)	Matched controls (*n* = 525)	*Z*/*χ*^2^	*p*-value	SMD
Age (years)	12.0(8.0, 14.0)	12.0(9.0, 14.0)	0.024	0.878	0.005
Gender, n(%)			0.000	1.000	0.000
Male	62(25.0%)	186(75.0%)			
Female	113(25.0%)	339(75.0%)			
BMI (kg/m^2^)	17.1(15.2, 20.5)	17.8(15.9, 21.1)	9.924	0.002	−0.288
CRP (mg/L)	0.3(0.0, 0.9)	0.2(0.0, 0.7)	1.267	0.261	−0.171
SF (μg/L)	62.1(33.6, 86.3)	63.6(44.2, 89.2)	4.377	0.036	0.176

### 3.2 Unconditional logistic regression analysis of SF and anemia risk

Table 2 presents the association between SF levels and anemia risk across progressively adjusted models. A consistent pattern was observed where the second (Q2) and fourth (Q4) quartiles of SF were associated with a significantly lower risk of anemia compared to the lowest quartile (Q1). In the fully adjusted model (Model 3), which controlled for age, sex, BMI, and CRP, participants in Q2 had an odds ratio (OR) of 0.39 (95% CI: 0.23–0.66, *p* = 0.001), and those in Q4 had an OR of 0.51 (95% CI: 0.30–0.85, *p* = 0.010). The association for the third quartile (Q3) was not statistically significant (OR = 0.66, 95% CI: 0.41–1.08, *p* = 0.099). Furthermore, a significant inverse linear trend was evident across the quartiles (*P* for trend = 0.011 in Model 3), with the strength of the trend increasing with greater adjustment for covariates.

**Table 2:** Adjusted odds ratio for anemia per quartile increase in SF.

Variable	Model 1		Model 2		Model 3	
	OR (95%CI)	*P***-**value	OR (95%CI)	*P***-**value	OR (95%CI)	*P***-**value
Q1	1.00		1.00		1.00	
Q2	0.43(0.26, 0.72)	0.001	0.41(0.25, 0.69)	0.001	0.39(0.23, 0.66)	0.001
Q3	0.72(0.45, 1.14)	0.161	0.67(0.41, 1.09)	0.110	0.66(0.41, 1.08)	0.099
Q4	0.60(0.37, 0.96)	0.033	0.56(0.34, 0.92)	0.022	0.51(0.30, 0.85)	0.010
*P* for trend		0.030		0.019		0.011

Odds ratios (ORs) and 95% confidence intervals (CIs) were derived from unconditional logistic regression models, conditioned on the matched sets. Model 1: SF. Model 2 + age,sex. Model 3 + CRP, BMI. Quartile ranges for SF (μg/L): Q1 ≤ 42.23; Q2 42.24–62.28; Q3 62.29–90.45; Q4 ≥ 90.46 (maximum 304.90).

### 3.3 Nonlinear dose–response relationship

**Figure 3:**
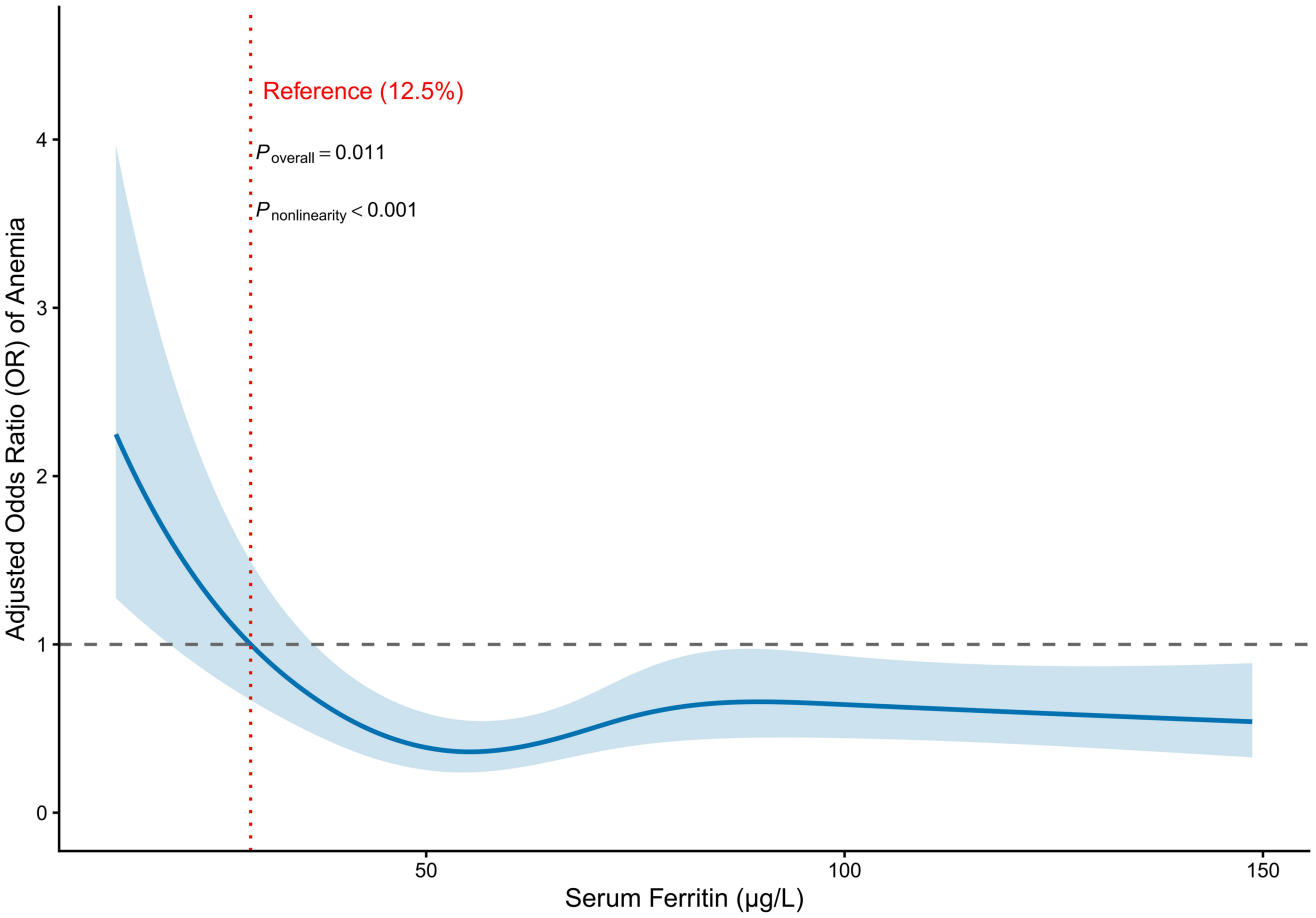
Dose–response relationship between SF and anemia.

The dose–response relationship between SF and anemia was modeled using restricted cubic splines. The restricted cubic spline curve (Figure 3) demonstrated a steep decline in anemia risk from low to moderate SF levels, followed by a plateau at higher levels, without a clear upward turn. The likelihood ratio test confirmed significant non-linearity (*χ*^2^ = 26.22, df = 4, *p* < 0.001).

### 3.4 Subgroup analysis and effect modification by sex

Table 3 displays the results of the sex-stratified analysis and the test for interaction between SF and sex on anemia risk. A statistically significant interaction was observed (*P* for interaction = 0.006), indicating that the association between SF and anemia differed meaningfully between males and females.

**Table 3:** Interaction analysis between SF and sex.

Variable	N (cases/controls)	OR (95% CI)	*P***-**value
SF (quartiles)			
Male	62/186		
Q1		1.00	
Q2		1.14(0.45, 2.92)	0.781
Q3		0.78(0.30, 2.07)	0.620
Q4		1.12(0.44, 2.84)	0.820
*P* for trend			0.983
Female	113/339		
Q1		1.00	
Q2		0.21(0.11, 0.44)	<0.001
Q3		0.75(0.42, 1.37)	0.352
Q4		0.36(0.18, 0.73)	0.004
*P* for trend			0.009
*P* for interaction			0.006

Stratified analysis by sex. Models adjusted for age, BMI, and CRP. Quartile ranges for SF (μg/L): Males – Q1 ≤ 51.15, Q2 51.16–70.43, Q3 70.44–95.35, Q4 ≥ 95.36 (max 299.10); Females – Q1 ≤ 37.30, Q2 37.31–57.18, Q3 57.19–87.22, Q4 ≥ 87.23 (max 304.90).

Stratified analysis revealed a distinct inverse association among females but not among males. In females, compared to the lowest quartile (Q1), the second (Q2: OR = 0.21, 95% CI: 0.11–0.44, *p* < 0.001) and fourth (Q4: OR = 0.36, 95% CI: 0.18–0.73, *p* = 0.004) quartiles of SF were associated with a significantly lower risk of anemia. A significant inverse linear trend was also present across quartiles in females (*P* for trend = 0.009). In contrast, no significant associations were found across any SF quartiles among males (all *p* > 0.05), nor was a significant trend observed (*P* for trend = 0.983).

### 3.5 Model performance

**Figure 4:**
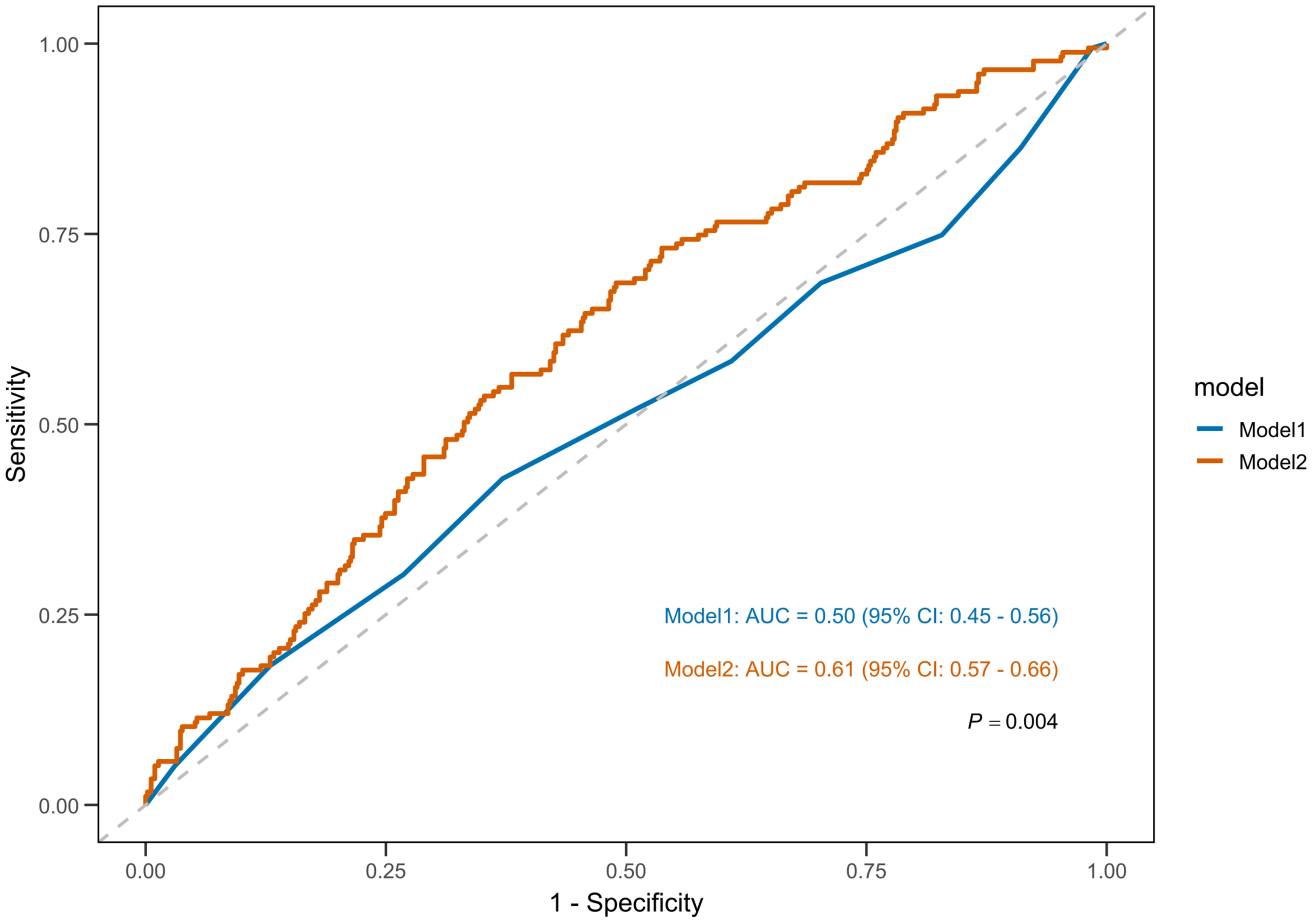
ROC curves for anemia prediction models.

**Figure 5:**
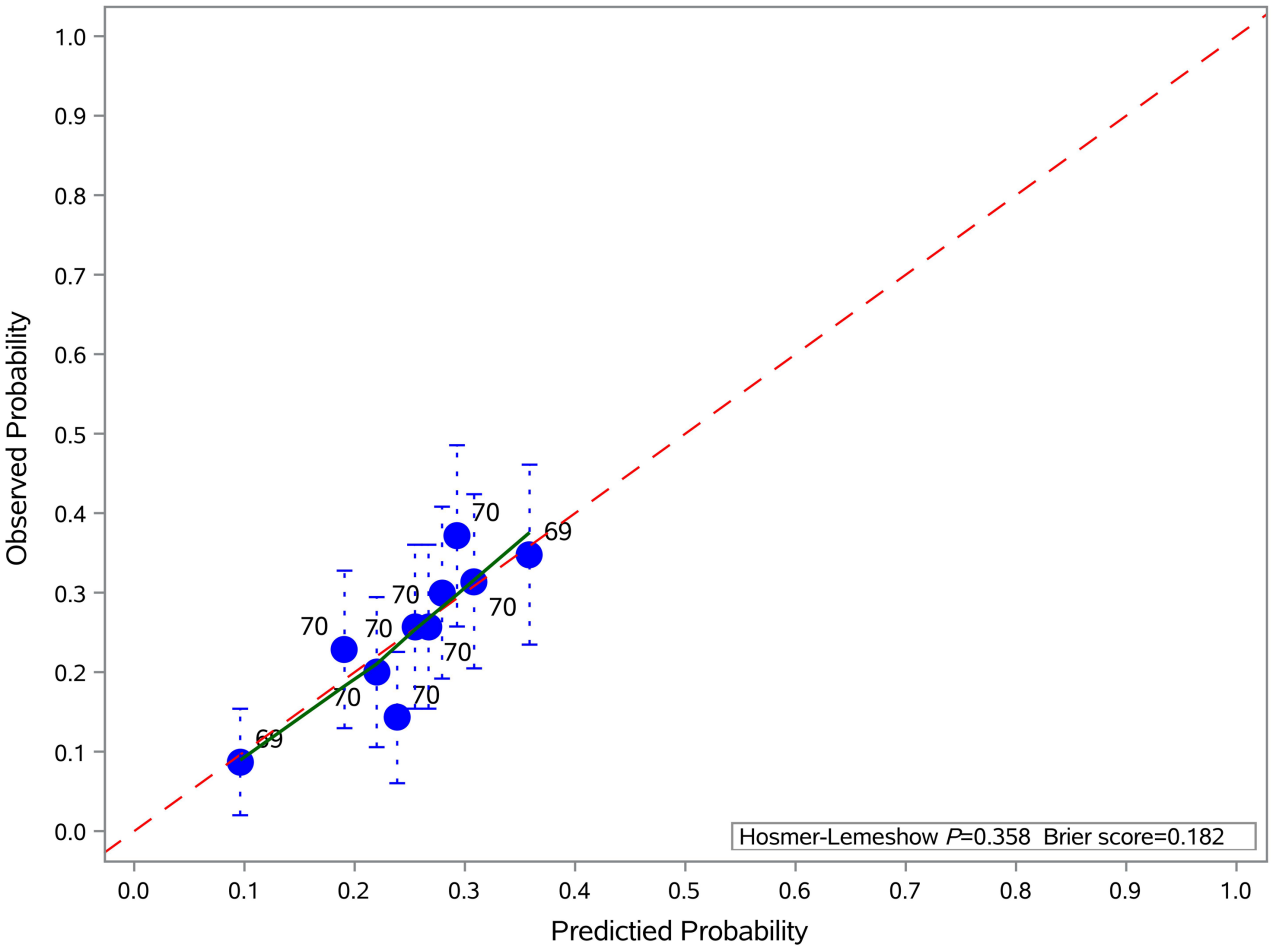
Model calibration curve.

Adding BMI and CRP to the base model (age+sex) significantly improved prediction, AUC increased from 0.50 (95%CI:0.45–0.56) to 0.61 (95%CI:0.57–0.66) (ΔAUC = 0.11, *p* = 0.004) (Figure 4). Calibration was excellent (Brier score = 0.182; Hosmer-Lemeshow *p* = 0.358) (Figure 5).

## 4 Discussion

The present matched case-control study investigated the association between SF levels and anemia risk in Chinese children and adolescents, utilizing rigorous 1:3 matching by age and sex to minimize potential confounding. Our principal findings demonstrate a significant non-linear inverse relationship between SF concentration and anemia risk, which persists after comprehensive adjustment for age, sex, BMI and CRP. Moreover, this association exhibits a pronounced sex-specific pattern, with strong and significant effects observed exclusively in females. Stratified analyses revealed a statistically significant interaction (*P* for interaction = 0.006), indicating genuine effect modification by sex.

After matching cases and controls at a 1:3 ratio, we identified significant differences in multiple nutritional biomarkers between anemic and non-anemic children. Notably, SF levels were substantially lower in anemic children compared to controls (median: 45.2 vs. 62.8 μg/L, *p* < 0.001), consistent with the well-established role of iron deficiency as a primary contributor to childhood anemia worldwide (15). This observation aligns with global estimates indicating that iron deficiency accounts for approximately 50% of all anemia cases in children, with particularly high burdens in low- and middle-income populations (16). A recent physiologically based study published in The Lancet Hematology by Addo et al. proposed new SF thresholds for iron deficiency of approximately 20 μg/L in children—substantially higher than the traditional WHO cutoff of 12 μg/L (17). This finding supports our observation that children in the lowest quartile (Q1) of SF, with values below approximately 40 μg/L, exhibited significantly elevated anemia risk, suggesting that the physiological threshold for compromised erythropoiesis may be higher than previously recognized (17). Furthermore, a large Indian study by Ghosh et al. demonstrated significant associations between SF and various health outcomes in adolescents, reinforcing the importance of iron status assessment in pediatric populations (18).

The non-linear dose–response relationship observed in our study extends the growing evidence that iron biomarkers may have optimal ranges for health. Similar non-linear associations between SF and health outcomes have been increasingly recognized. A genome-wide meta-analysis by Moksnes et al. published in Communications Biology demonstrated a non-linear relationship between serum iron and all-cause mortality, with protective effects at moderate levels and attenuated benefits at extremes (10). More specifically, a systematic review and meta-analysis by Yu et al. in Frontiers in Nutrition examined the dose–response relationship of SF with metabolic outcomes and identified that each 50 μg/L increase in SF was associated with a 1.15-fold higher risk in males and 1.50-fold higher risk in females, suggesting sex-specific non-linear patterns (19). Our restricted cubic spline analysis, with formal testing confirming significant non-linearity (*p* < 0.001), revealed that anemia risk decreased from Q1 to Q2, plateaued around Q3, and remained reduced at Q4. This pattern suggests that while moderate increases in SF confer substantial protection, the incremental benefit diminishes at higher levels—a finding consistent with the concept of optimal iron storage ranges rather than simple linear dose–response. The centrality of SF as the preferred biomarker for assessing iron status in children is further supported by evidence that reliance on serum iron alone can lead to substantial misclassification. A study by Sezgin et al. found that iron-replete children had 2.59-fold greater odds of being diagnosed with iron deficiency if evaluated based on low serum iron results rather than SF, underscoring the importance of using SF for accurate assessment in pediatric populations (20).

The attenuated inverse association at the highest SF quartile (Q4: OR = 0.51, 95% CI: 0.30–0.85) compared to Q2 (OR = 0.39, 95% CI: 0.23–0.66) warrants careful interpretation. In pediatric populations, markedly elevated SF may not uniformly represent superior iron status. SF is a positive acute-phase reactant that can be elevated by underlying subclinical inflammation, which itself contributes to anemia of inflammation through hepcidin-mediated iron sequestration (21). Our adjustment for C-reactive protein partially addresses this concern, but residual confounding from unmeasured inflammatory markers may persist. This aligns with recommendations from the Biomarkers Reflecting Inflammation and Nutritional Determinants of Anemia (BRINDA) project, which emphasizes the necessity of adjusting SF for inflammation when assessing iron status in populations (22). Studies have shown that failure to account for inflammation can lead to overestimation of iron stores in individuals with elevated acute-phase proteins (23). Therefore, the Q4 group in our study likely comprises a mixture of truly iron-replete individuals and those with inflammation-distorted iron homeostasis, accounting for the diminished inverse association observed at higher SF concentrations.

The most striking finding is the significant sex-specific association. The strong, graded inverse relationship in females (*P* for trend = 0.009), contrasted with the null association in males (*P* for trend = 0.983), underscores fundamental differences in iron physiology during adolescence. This observation is consistent with well-documented sex disparities in iron metabolism. A study by Shoemaker et al. among youth athletes reported a markedly higher prevalence of iron depletion in females (86%) compared to males (65%), with females showing consistently poorer iron status across all measured biomarkers. The authors linked this divergence to pubertal development, noting that males exhibited higher SF concentrations while females were more biologically mature (24). Consistent with this, a 2024 study from Ghana by Amponsah-Doku et al. demonstrated pronounced sex differences in iron status among adolescents, with median SF levels significantly lower in females (18.06 ng/mL) compared to males (51.23 ng/mL) (*p* < 0.001) (25). The authors observed that this disparity persisted even after accounting for dietary intake, suggesting that biological factors—including menstrual blood loss and hormonal regulation of iron metabolism—play a predominant role in shaping iron status during adolescence. A comprehensive 2021 review by Farag et al. further emphasized that sex-specific differences in iron requirements and metabolism emerge during puberty and persist throughout the reproductive years, driven by the interplay of genetic, hormonal, and physiological factors beyond simple dietary inadequacy. Our results empirically confirm that SF is a potent biomarker for anemia risk specifically in adolescent girls, who face heightened iron demands due to growth acceleration and menstrual onset. In males, whose iron requirements are comparatively stable, anemia in this age group may have more diverse etiologies—such as other nutritional deficiencies (e.g., vitamin B12, folate) or underlying chronic conditions—diminishing the relative explanatory power of SF alone (26). This sex-specific pattern has also been observed in other populations. A study from rural South India reported that anemic adolescents had significantly lower serum iron and higher total iron-binding capacity, with SF levels markedly lower in anemic boys compared to anemic girls, further reinforcing the fundamental role of sex in shaping iron status during adolescence (27). These differences likely arise from differential regulation of iron metabolism by pubertal hormones, with androgens promoting erythropoiesis and iron utilization while estrogens may influence hepcidin expression (28, 29).

Intriguingly, our analysis revealed a non-linear pattern in females that was not observed in males. In females, compared to the lowest quartile (Q1), the second (Q2: OR = 0.21, 95% CI: 0.11–0.44, *p* < 0.001) and fourth (Q4: OR = 0.36, 95% CI: 0.18–0.73, *p* = 0.004) quartiles were both associated with significantly lower anemia risk, while Q3 showed a non-significant intermediate association. This pattern may reflect the complex interplay between iron stores, menstrual status, and hormonal influences. Pubertal hormones, particularly estrogens and androgens, differentially regulate hepcidin expression and iron absorption (30). A study by Oh et al. in Korean children and adolescents demonstrated that SF reference values differ significantly by sex and pubertal stage, with boys showing progressive increases through adolescence while girls plateau after menarche (31). The mechanisms underlying these differences may involve prolactin enhancement of luteinizing hormone effects on mesenchymal stromal cells and subsequent testosterone synthesis, ultimately elevating SF by inhibiting iron-modulating hormone synthesis (32, 33)^.^

The addition of BMI and CRP to a base model containing age and sex significantly improved anemia prediction, as evidenced by the increase in AUC from 0.50 to 0.61 (ΔAUC = 0.11, *p* = 0.003). This suggests that SF measurement could enhance the identification of children at high risk of anemia, particularly when interpreted alongside inflammatory markers. The model also demonstrated excellent calibration (Brier score = 0.182; Hosmer-Lemeshow *p* = 0.358), indicating good agreement between predicted and observed outcomes. These findings support the potential clinical utility of incorporating SF into pediatric anemia risk assessment, though the modest AUC (0.61) suggests that other unmeasured factors also contribute importantly to anemia risk.

Given the substantial burden of both iron deficiency and anemia among Chinese children, our findings support integrated nutritional interventions that consider both iron status and inflammation, with particular attention to adolescent girls. A recent meta-analysis by Dhanvijay et al. comparing daily versus alternate-day oral iron supplementation found comparable efficacy for treating iron deficiency anemia, with both regimens producing significant improvements in hemoglobin (34). Since our results indicate that SF is a relevant biomarker primarily for females, targeted screening and intervention programs focusing on adolescent girls may yield greater public health impact. Furthermore, given the potential confounding by inflammation suggested by our Q4 findings, interventions should ideally be coupled with inflammation assessment to avoid misclassifying inflammation-elevated SF as adequate iron stores.

Several limitations should be considered. First, the observational design precludes causal inference, despite the supportive dose–response gradient and biological plausibility. Second, although we used a DAG to adjust for key confounders (age, sex, BMI, CRP), residual confounding from unmeasured or imperfectly measured factors (e.g., detailed dietary iron intake, helminth infections, genetic hemoglobinopathies, hepcidin levels) may persist. Notably, while we adjusted for CRP, the lack of data on other inflammatory cytokines limits our ability to fully disentangle inflammation-confounded iron status. Furthermore, we did not have information on menstrual status or pubertal stage in female participants, which are important potential confounders for the observed sex-specific associations, particularly in post-menarcheal adolescents. Third, reliance on a single measurement of SF and CRP may not capture long-term status and introduces variability. Fourth, our sample was drawn from rural Chinese counties, which may limit generalizability to urban populations or other ethnic groups. Fifth, the male subgroup contained a relatively small number of anemia cases (*n* = 62), which reduced statistical power and resulted in wide confidence intervals; therefore, the absence of a statistically significant association in males should not be interpreted as definitive evidence of no effect. Sixth, we used BMI as the sole proxy for nutritional status, whereas more comprehensive anthropometric indices (e.g., weight-for-age, height-for-age, weight-for-height) would provide a fuller assessment of undernutrition and stunting in pediatric populations. Future studies should incorporate these measures.

In conclusion, this matched case–control study revealed a significant non-linear inverse association between SF levels and anemia risk among children and adolescents in southwest China, with a pronounced sex-specific effect demonstrating strong associations in females but not in males. The non-linear dose–response relationship, with optimal protection at moderate SF levels and attenuated benefit at higher levels after adjustment for CRP, underscores the complexity of iron metabolism and its relationship to anemia. These findings support the integration of SF measurement with inflammatory markers in pediatric anemia assessment and highlight the importance of sex-specific approaches to screening and intervention. Future longitudinal studies and randomized trials incorporating detailed menstrual history, hepcidin measurement, and comprehensive inflammatory panels are warranted to confirm these associations and elucidate the underlying mechanisms, particularly the observed sex-specific effects.

## 5 Conclusion

In conclusion, this study demonstrates that the relationship between SF and anemia in children and adolescents is characterized by a non-linear dose–response pattern and is profoundly modified by sex, being a relevant biomarker primarily for female adolescents. These insights advocate for a more nuanced, context-aware, and individualized approach to diagnosing and managing pediatric anemia, integrating inflammation status and sex-specific considerations.

## Data Availability

The raw data supporting the conclusions of this article will be made available by the authors, without undue reservation.
